# Current and preservice teachers' views and beliefs regarding martial arts and the inclusion of martial arts in Australian school settings: A cross‐sectional study

**DOI:** 10.1002/hsr2.1351

**Published:** 2023-06-16

**Authors:** Louis Burt, Nicholas Riley, Narelle Eather

**Affiliations:** ^1^ College of Human and Social Futures University of Newcastle Callaghan New South Wales Australia; ^2^ Centre for Active Living and Learning (CALL) Callaghan New South Wales Australia

**Keywords:** combat sport, curriculum, preservice teachers, questionnaire, well‐being

## Abstract

**Purpose:**

This study investigates views and beliefs of current and preservice teachers regarding martial arts (MA) and the inclusion of martial arts in schools.

**Methods:**

Participants completed an anonymous, 28‐item questionnaire made available online via Qualtrics (August–November 2020). Data was analysed using SPSS software to compare mean scores by sex, and between qualified teachers and preservice teachers. Qualitative data in the form of quotes was drawn upon and used to complement the quantitative results.

**Results:**

Results indicate teachers and preservice teachers view MA as a worthwhile and beneficial activity for school‐aged students, and support the inclusion of MA into school settings.

**Conclusion:**

These findings may be useful to inform policy and practice in schools, and the development of teacher education programs, professional development courses, and school‐based education programs utilizing MA to meet physical education learning outcomes.

## 1 INTRODUCTION

“Martial arts’”(MA) is a collective term that encompasses the multitude of combat sports, self‐defense, and hand‐to‐hand combat systems developed and implemented around the world.^1^ Studies of MA participation have identified a wide range of benefits to school‐aged children. Physiological improvements include increased oxytocin levels,^2^ increased flexibility, muscular fitness, and balance.^3^, ^4^ Additionally, emerging evidence indicates that MA may facilitate positive change in psychological,^5^ cognitive,^6^ and academic outcomes.^7^ Internationally, education systems recognize the benefits of MA, with countries such as the USA having school wrestling teams and associated scholarships, while Budo (an umbrella term for traditionalist Japanese MA) is a mandatory element of junior high‐school physical education in Japan (Chambers and colleagues).

Despite evidence of the positive benefits, some researchers warn against increased aggression levels and antisocial behavior among participants (when fighting techniques are the sole focus, rather than a holistic approach.^8^, ^9^ Furthermore, fighting skills and violence are often glorified and propagated in movies, television shows and other popular culture,^10^ creating some unrealistic and/or untrue perceptions of MA as a collective. Thus, the incorporation of MA into school settings remains a challenge, partially due to school administrators' and teachers' safety concerns, and their perceptions of MA, and/or a lack of teacher training to deliver MA in an effective manner which meets curriculum objectives.^11^

Within Australia, the New South Wales (NSW), Australian Capital Territory (ACT), South Australia (SA), Victoria (VIC) Department(s) of Education each currently provide specific safety guidelines and procedures for the teaching of MA within school contexts (NSW Government).^12^ The guidelines suggest that MA is acceptable for implementation in both Primary and Secondary schools (assuming the safety guidelines are followed), but it is unclear whether teachers in these states are aware of these guidelines, and data confirming if and when MA is used in schools does not exist.

Furthermore, numerous outcomes in both the current Australian Health and Physical Education curriculum, and NSW Personal Development, Health and Physical Education curriculum lend themselves to MA.^13^, ^14^ For example, traditional MA often incorporate ethical and social education, through methods such as meditation and philosophy, discussion, and consistent etiquette expectations (bowing to each other, showing respect to higher level students and instructors, and respecting equipment) in addition to physical training.^8^ A number of outcomes from the afore mentioned syllabi, associated with physical activity, align with the foundations on which MA builds on, such as “adapting movement skills in a variety of contexts.”^13^ For example, in grappling and wrestling, one must constantly “adapt movements and techniques” as not to be predictable and easy to counter. A student of MA must also “propose, apply, and assesses solutions to movement challenges.”^13^ For example: in competition or practice, if a certain technique is not effective on their opponent/training partner, they must quickly think of a solution or new technique to perform that will allow for a solution. In relation to movement patterns, a MA student may “create, perform and analyse” their own *kata* (sequence of movements, similar to a dance routine).^15^

Since mathematical literacy was first assessed as a major domain in 2003, Australia's average score for mathematical literacy has declined by 33 points (equivalent to more than 1 year of schooling) (Thomson and colleagues). Australia is also under‐performing in relation to physical activity, ranking at a tied 32nd place out of 49 countries for their “Overall Physical Activity” grade.^16^ A systematic review by Watson et al.^17^ concluded that classroom‐based physical activity interventions may offer practical, cost‐effective, strategies to increase physical activity levels as well as academic‐related outcomes, particularly for improving on‐task behavior, and consequentially reducing off‐task classroom behavior and selective attention.^17^ The novel work of Riley et al.^18^ investigated this claim, and found that embedding movement‐based learning in mathematics lessons had a significant positive effect on children's engagement and enjoyment, without conceding the quality of learning.^19^ A more recent study, conducted by Burt et al.^7^ found that a school‐based martial arts program which embedded mathematics concepts into martial arts lessons had significant positive effects on physical and academic outcomes. Additionally, feasibility assessments in this study returned highly positive results relating to perceived benefits and enjoyment, gained from both student participants and their class teachers.^7^ Due to an increasingly defined curriculum and increased accountability for teachers, current teachers are experiencing the overcrowding of Australian school curriculums.^20^ This presents a need for innovative ways to help teachers meet demands in a way which is physically and cognitively engaging for children. The unique and diverse characteristics of MA may present teachers with the ability to integrate MA into physical education, mathematics or literacy programs, while increasing activity levels of Australian school‐aged children, whose activity levels remain lower than the recommended levels.

In summary, active participation in MA has been shown to provide a wide range of benefits to school‐aged children, including psychological,^5^ cognitive,^6^ and academic outcomes,^7^ but remains a nonexistent or fringe activity in many schools around Australia. This brings to the fore a need to investigate the views and beliefs of current and preservice teachers regarding MA and the inclusion of MA in school settings.

## 2 MATERIALS AND METHODS

### 2.1 Survey

The research team consisting of four qualified teachers (two of whom are highly experienced MA instructors and two highly experienced PDHPE teachers), developed a purpose‐designed questionnaire (for access to full questionnaire, email lead author) to target the study aims. After both university and NSW State Education Research Applications Process (SERAP) ethics approval was granted, the survey was then transferred online *to Qualtrics* and made available for participants between the August 26 and November 23, 2020. The survey consisted of 9 demographic questions presented in a multiple‐choice format, and 19 topic‐specific questions presented in a 5‐point Likert scale format, and two open‐ended questions. The survey was separated into the following nine sections:

1. List of definitions
2. Demographic information

a. General demographic information (gender; age; school type; MA experience; current year of undergraduate study [if applicable], area of education studied [or studying])
b. Qualified (and practicing) teachers (section only accessible to these participants) (e.g., years teaching experience; public or private school; time allocated to practical PE in their classroom each week; time allocated to sport in their classroom each week)

3. School climate (this was only accessible to qualified practicing teachers) (3 questions, e.g., *To what extent do you believe that behavioral issues (e.g., bullying, violence, disrespect, vandalism) is an issue in your school?*)
4. Beliefs (12 questions, e.g., *I believe the NSW K‐10 PDHPE Syllabus lends itself to the incorporation of martial arts*)
5. Perceived benefits for your students (10 questions, e.g., *Martial arts training can help increase energy levels in children and adolescents*)
6. Enjoyment (three questions, e.g., *I believe children and/or adolescents in the class(es) I teach would enjoy participating in martial arts training*)
7. Future plans (six questions, e.g., *In the future, I would be interested in learning how to incorporate martial arts in a classroom setting*)
8. Martial arts topics most beneficial for school students (11 topics, e.g., *Learning how to Punch*)
9. Concerns and suggestions (qualitative)

### 2.2 Recruitment and participants

Invitations were sent to principals of independent and public schools (*n* = 22) in the Greater Hunter region (NSW, Australia). The questionnaire was also shared to the online student learning platform for 16 undergraduate physical education teaching courses for the programs: Bachelor of Education (Secondary) (PDHPE); Bachelor of Education (Early Childhood and Primary); Bachelor of Education (Primary), at the University of Newcastle, Australia. The questionnaire was also advertised on six Facebook sites designed for Australian teachers, and undergraduate teachers to network and communicate (after consent was received from the page administrators). Additionally, each member of the research team posted the questionnaire on their personal social media pages (e.g., twitter, Facebook).

### 2.3 Consent

All participants needed to provide informed consent to complete the questionnaire.

### 2.4 Analysis

Quantitative data was analysed using IBM SPSS Statistics version 22 (IBM Corp) and descriptive statistics were explored (means [M], standard deviations [SD]), One‐way ANOVA was used to determine if differences between study outcomes existed between demographic groups. Qualitative data in the form of quotes was drawn upon used to complement the quantitative results.

## 3 RESULTS

### 3.1 Descriptive statistics

The descriptive statistics are summarized in Table 1. A total of 97 current (37.1%; *n* = 36) and preservice teachers (62.9%; *n* = 61) completed the survey between the dates of August 26 and November 23, 2020; 63.9% (*n* = 62) female, 34% (*n* = 33) male. The most common age group was 18 to 24‐year‐old, representing 62.9% (*n* = 61) of participants. The most represented teacher or undergraduate studying to become such, in this study sample was PDHPE Teachers (38%; *n* = 35). Most participants 66% (*n* = 64) indicated that they had never participated in any form of MA, and only 3.1% (*n* = 3) indicated that they had consistently participated in MA for *5+ years*. When asked if they considered themselves to be physically fit, the majority of participants answered either “somewhat agree” (45.4%; *n* = 44), or “strongly agree” (23.7%; *n* = 23).

**Table 1:** Teachers' rankings of barriers for teaching martial arts their schools.

Rank	Field	Mean	Standard deviation
1	Teachers' lack of knowledge/skills in teaching martial arts	4.46	1.05
2	Not enough training opportunities for teachers	4.11	1.08
3	Lack of interest/willingness of teachers to teach martial arts	3.82	1.26
4	No time in teachers schedule to explore opportunities for teaching new types of physical education	3.79	1.11
5	Insufficient equipment for students to use	3.68	1.26
6	Insufficient equipment for teachers to use	3.68	1.31
7	Not enough variety of equipment	3.57	1.27
8	Teachers' lack of knowledge/skills in teaching physical education	3.54	1.40
9	Damaged and/or poor‐quality equipment	3.36	1.23
10	Out‐dated equipment	3.36	1.23
11	Problems scheduling time in appropriate school areas (e.g., basketball court, fields, etc) for each class	3.11	1.35

*Note*: Question: Do you consider any of the following a barrier for teaching martial arts in your school. 1 = strongly disagree (is not a barrier); 2 = disagree; 3 = unsure; 4 = somewhat agree; 5 = strongly agree (is a barrier).

### 3.2 Qualified (and practicing) teachers

Survey participants were predominantly from public schools, representing 75% of the sample—indicative of the larger proportion of teachers in the government sector, compared to independent and catholic schools—and teachers of Stage 3 (*n* = 20/36). On average, teaching experience was 3.63 years (SD = 2.27; *n* = 16), with only three participants stating that they had been working for 6—10 years, or longer than 10 years.

### 3.3 School climate

Of the 28 practicing teachers who completed this section, 60.7% either “somewhat agree[d]” (21.4%; *n* = 6), or “strongly agree[d]” (39.3%; *n* = 11) that *behavioral issues (e.g., bullying, violence, disrespect, vandalism)* [are] *an issue in* [their] *school*. From the same 28 teachers, 82% either “somewhat agree[d]” (42.9%; *n* = 12), or “strongly agree[d]” (39.3%; *n* = 11) that these behavioral issues *impact negatively on learning in the classroom*. Additionally, teachers were given a list of potential *barriers for teaching martial arts in* [their] *school* and asked to rate each of them on a 5‐point Likert scale as being, or not being a barrier. These barriers were ranked as shown in Table 1.

### 3.4 Beliefs and attitudes toward martial arts; perceived benefits of martial arts training; future plans; most beneficial martial arts topics, as perceived by participants

Results from practicing teachers and preservice teachers indicate a low perception of Government recognition of MA in NSW schools, with mean a participant score of 2.94 out of 5 (*N* = 88; SD = 0.864) for item Q17.6 (see Table 2). Most participants reported that MA training can provide a wide range of benefits to their students, including improved muscular fitness (Mean = 4.34; *N* = 86; SD = 0.713), improved aerobic fitness (Mean = 4.22; *N* = 86; SD = 0.773), and improved mental health (Mean = 4.20; *N* = 86; SD = 0.733). The vast majority of participants “would support the inclusion of MA into the curriculum” (Mean = 4.26; *N* = 88; SD = 0.864). Most participants 66% (*n* = 64) have never participated in any form of MA training, and would not feel *comfortable teaching this* (Mean = 3.15; *N* = 89; SD = 1.284).

**Table 2:** Comparison of means—Teachers and preservice teachers.

Beliefs											
Question	Field	Teacher (*n* = 27)			Preservice teacher (*n* = 61)			Total (*n* = 88)			
		Mean	*N*	SD	Mean	*N*	SD	Mean	*N*	SD	Significance
Q17.1	I believe martial arts training has a place in a primary school setting	3.89	27	1.05	3.62	61	0.80	3.70	88	0.89	0.196
Q17.2	I believe martial arts training has a place in a high school setting	4.00	27	0.96	3.87	61	0.81	3.91	88	0.85	0.509
Q17.3	I think all primary school‐aged students should have the opportunity to try martial arts training	4.11	27	0.97	4.00	61	0.75	4.03	88	0.82	0.562
Q17.4	I think all high school‐aged students should have the opportunity to try martial arts training	4.30	27	0.91	4.18	61	0.56	4.22	88	0.69	0.468
Q17.5	I believe the NSW K‐10 PDHPE syllabus lends itself to the incorporation of martial arts	3.41	27	1.05	3.46	61	0.96	3.44	88	0.98	0.821
Q17.6	I believe the NSW Department of Education promotes martial arts as an acceptable in‐school sport	2.33	27	1.11	3.21	61	0.88	2.94	88	1.03	0.000
Q17.7	Incorporating physical activity of any kind into the instruction of Key Learning Areas (KLA's) such as Mathematics or English could be beneficial for students	4.33	27	0.92	4.11	61	0.88	4.18	88	0.89	0.291
Q17.8	Incorporating martial arts training into the instruction of KLA's such as Mathematics or English could be beneficial for students	3.89	27	1.01	3.62	61	1.04	3.70	88	1.03	0.266
Q17.9	If self‐defense, or a martial art (of any kind) was introduced to the curriculum, I would support this	4.33	27	1.04	4.23	61	0.78	4.26	88	0.86	0.606
Q17.10	If self‐defense, or a martial art of any kind was introduced to the curriculum, I would be confident teaching this	2.71	28	1.49	3.34	61	1.14	3.15	89	1.28	0.031
Q17.11	A school‐based martial arts program that addresses PDHPE syllabus outcomes is appealing	4.07	28	1.18	3.92	61	0.82	3.97	89	0.95	0.481
Q17.12	A school‐based martial arts program that addresses other KLA syllabus outcomes is appealing	4.07	28	1.12	3.87	61	0.81	3.93	89	0.92	0.335

Perceived benefits of martial arts training (Each statement began with “0*Martial arts training can…”0)*											
Question	Field	Teacher			Preservice teacher			Total			
		Mean	*N*	SD	Mean	*N*	SD	Mean	*N*	SD	Significance
Q18.1	…Help increase energy levels in children and adolescents	4.31	26	1.09	4.10	60	0.60	4.16	86	0.78	0.259
Q18.2	…Improve aerobic fitness in children and adolescents	4.27	26	1.15	4.20	60	0.55	4.22	86	0.77	0.705
Q18.3	…Improve muscular fitness in children and adolescents	4.58	26	0.90	4.23	60	0.59	4.34	86	0.71	0.039
Q19.1	…Improve mental health in children and adolescents	4.46	26	0.91	4.08	60	0.62	4.20	86	0.73	0.027
Q19.2	…Improve motivation to exercise in children and adolescents	4.27	26	1.00	4.12	60	0.69	4.16	86	0.80	0.417
Q19.3	…Help children and adolescents feel like they can persevere and accomplish more	4.42	26	0.90	4.12	60	0.69	4.21	86	0.77	0.090
Q19.4	…Help children and adolescents feel more motivated at school	4.23	26	0.95	3.95	60	0.81	4.03	86	0.86	0.166
Q19.5	…Help children and adolescents feel more motivated at school	4.19	26	0.69	3.97	60	0.76	4.03	86	0.74	0.197
Q19.6	…Help children and adolescents get along better with other people in their life (e.g., family, classmates, friends)	4.12	26	0.77	3.78	60	0.83	3.88	86	0.82	0.084
Q19.7	…Address violent/antisocial behavior in schools	4.04	26	0.66	3.92	60	0.89	3.95	86	0.83	0.533

Attitudes toward teaching martial arts											
Question	Field	Teacher			Preservice teacher			Total			
		Mean	*N*	SD	Mean	*N*	SD	Mean	*N*	SD	Significance
20.1	I believe children and/or adolescents in the class(es) I teach would enjoy participating in martial arts training	4.04	26	0.96	4.02	60	0.73	4.02	86	0.80	0.908
20.2	I believe children and adolescents in general would enjoy participating in martial arts training	4.19	26	0.90	4.1	60	0.71	4.13	86	0.76	0.610
20.3	I would enjoy teaching martial arts to students	3.46	26	1.36	3.88	60	0.90	3.76	86	1.07	0.094

Future plans											
Question	Field	Teacher			Preservice teacher			Total			
		Mean	*N*	SD	Mean	*N*	SD	Mean	*N*	SD	Significance
Q22.1	In the future, I would be interested in learning how to incorporate martial arts in a classroom setting	4.12	26	1.03	4.00	59	0.85	4.04	85	0.91	0.591
Q22.2	I would be interested in attending professional development/accreditation to learn how to teach basic martial arts techniques to my students	3.92	26	1.32	3.92	59	0.84	3.92	85	1.00	0.974
Q22.3	In the future, I would be interested in a martial arts‐based program that also improved NSW KLA syllabus outcomes	4.27	26	0.96	3.78	59	0.77	3.93	85	0.86	0.014
Q22.4	If martial arts were introduced into the NSW curriculum, I would outsource the instruction, rather than teach it myself	3.65	26	1.09	3.63	59	0.89	3.64	85	0.95	0.906
Q22.5	In the future I would be comfortable incorporating martial arts in my own classroom	3.58	26	1.14	3.66	59	0.86	3.64	85	0.95	0.709
Q22.6	In the future I plan on participating/training (or continuing) in some form of martial art (e.g., Boxing, Kickboxing, Wrestling, Jujitsu, Karate, Taekwon‐Do etc)	3.12	26	1.48	3.39	59	1.13	3.31	85	1.24	0.352

Most beneficial martial arts topics for children and adolescents											
Question	Field	Teacher			Preservice teacher			Total			
		Mean	*N*	SD	Mean	*N*	SD	Mean	*N*	SD	Significance
Q23.1	Movement/parkour type activities (e.g., rolling, jumping, vaulting, falling safely)	4.60	25	0.58	4.05	59	0.66	4.21	84	0.68	0.000
Q23.2	Learning how to dodge and block attacks	4.36	25	0.64	4.24	59	0.65	4.27	84	0.65	0.430
Q23.3	Learning how to punch	3.68	25	1.11	3.75	59	1.08	3.73	84	1.08	0.800
Q23.4	Learning how to kick	3.76	25	1.13	3.73	59	1.03	3.74	84	1.05	0.902
Q23.5	Grappling/wrestling (e.g., how to safely escape if tackled or grabbed)	4.20	25	0.91	3.98	59	0.90	4.05	84	0.90	0.317
Q23.6	Teamwork/team building activities among classmates	4.72	25	0.54	4.44	59	0.68	4.52	84	0.65	0.071
Q23.7	Wellbeing/ethical discussions at the end of each session, focussing on topics such as respect, integrity, self‐control and so forth	4.68	25	0.63	4.42	59	0.62	4.50	84	0.63	0.089
Q23.8	Olympic martial arts: Judo, Taekwon‐Do and Karate	3.92	25	1.00	3.75	59	1.06	3.80	84	1.04	0.485
Q23.9	Learning katas/forms/poomsae (set movement routines)	3.84	25	1.11	3.64	59	1.05	3.70	84	1.06	0.443
Q23.10	Meditation/mindfulness	4.48	25	1.01	4.32	59	0.84	4.37	84	0.89	0.460
Q23.11	Learning how to use various traditional weapons (e.g., swords, Bo staff, Kali sticks)	2.88	25	1.48	3.10	59	1.26	3.04	84	1.32	0.485

Abbreviations: *N*, number of responses; SD, standard deviation.

Many participants, however, indicated they *would enjoy teaching* the subject matter to their students (Mean = 3.76, *N* = 86; SD = 1.073), and *would be interested in learning how to incorporate martial arts into a classroom setting* (Mean = 4.04; *N* = 85; SD = 0.096), and *would be interested in attending professional development/accreditation to learn how to teach basic martial arts techniques to [their] students* (Mean = 3.92; *N* = 85; SD = 1.003).

#### 3.4.1 Comparison of means—Teachers and preservice teachers

##### Beliefs

As shown in Table 3, there were only two questions with statistically significant differences between groups. The first being Q17.6*: I believe the NSW Department of Education promotes martial arts as an acceptable in‐school sport* (*p* = 0.000). This was the lowest mean score for both groups. Preservice teacher participants rated higher on this question (Teachers = 2.33; Preservice teachers = 3.21), indicating they were more likely to have a higher perception of Government recognition of MA in NSW schools. The second statistically significant difference was seen in Q17.9: *If self‐defense, or a martial art (of any kind) was introduced to the curriculum, I would support this*. (*p* = 0.031). Both groups, however scored this section highly to very highly, having both groups rating >4.2 out of 5. This had the highest mean score for both groups. *Perceived benefits of martial arts training*.

**Table 3:** Comparison of means—Sex.

Beliefs											
Question	Field	Female			Male			Total			
		Mean	*N*	SD	Mean	*N*	SD	Mean	*N*	SD	Significance
Q17.1	I believe martial arts training has a place in a primary school setting	3.59	56	0.85	4.00	30	0.79	3.70	88	0.89	0.031
Q17.2	I believe martial arts training has a place in a high school setting	3.86	56	0.82	4.10	30	0.76	3.91	88	0.85	0.182
Q17.3	I think all primary school‐aged students should have the opportunity to try martial arts training	4.04	56	0.81	4.17	30	0.65	4.03	88	0.82	0.446
Q17.4	I think all high school‐aged students should have the opportunity to try martial arts training	4.16	56	0.73	4.33	30	0.61	4.22	88	0.69	0.273
Q17.5	I believe the NSW K‐10 PDHPE syllabus lends itself to the incorporation of martial arts	3.50	56	0.89	3.47	30	1.04	3.44	88	0.98	0.877
Q17.6	I believe the NSW Department of Education promotes martial arts as an acceptable in‐school sport	3.07	56	0.97	2.77	30	1.10	2.94	88	1.03	0.189
Q17.7	Incorporating physical activity of any kind into the instruction of Key Learning Areas (KLA's) such as Mathematics or English could be beneficial for students	4.21	56	0.83	4.27	30	0.83	4.18	88	0.89	0.780
Q17.8	Incorporating martial arts training into the instruction of KLA's such as Mathematics or English could be beneficial for students	3.64	56	0.96	3.93	30	1.05	3.70	88	1.03	0.199
Q17.9	If self‐defense, or a martial art (of any kind) was introduced to the curriculum, I would support this	4.14	56	0.96	4.47	30	0.63	4.26	88	0.86	0.100
Q17.10	If self‐defense, or a martial art of any kind was introduced to the curriculum, I would be confident teaching this	3.05	56	1.26	3.32	31	1.35	3.15	89	1.28	0.355
Q17.11	A school‐based martial arts program that addresses PDHPE syllabus outcomes is appealing	3.84	56	0.99	4.23	31	0.85	3.97	89	0.95	0.069
Q17.12	A school‐based martial arts program that addresses other KLA syllabus outcomes is appealing	3.82	56	0.97	4.19	31	0.75	3.93	89	0.92	0.069

**Perceived benefits of martial arts training (each statement began with** ***Martial arts training can…)***											
**Question**	**Field**	**Female**			**Male**			**Total**			
		**Mean**	***N***	**SD**	**Mean**	***N***	**SD**	**Mean**	***N***	**SD**	Significance
18.1	…Help increase energy levels in children and adolescents	4.06	53	0.80	4.35	31	0.76	4.16	86	0.78	0.095
18.2	…Improve aerobic fitness in children and adolescents	4.13	53	0.83	4.39	31	0.67	4.22	86	0.77	0.150
18.3	…Improve muscular fitness in children and adolescents	4.15	53	0.74	4.68	31	0.54	4.34	86	0.71	0.001
19.1	…Improve mental health in children and adolescents	4.13	53	0.79	4.32	31	0.65	4.20	86	0.73	0.258
19.2	…Improve motivation to exercise in children and adolescents	4.09	53	0.84	4.32	31	0.70	4.16	86	0.80	0.205
19.3	…Help children and adolescents feel like they can persevere and accomplish more	4.13	53	0.83	4.39	31	0.62	4.21	86	0.77	0.142
19.4	…Help children and adolescents feel more motivated at school	3.98	53	0.89	4.16	31	0.82	4.03	86	0.86	0.359
19.5	…Help children and adolescents feel more motivated at school	3.96	53	0.78	4.19	31	0.65	4.03	86	0.74	0.170
19.6	…Help children and adolescents get along better with other people in their life (e.g., family, classmates, friends)	3.85	53	0.86	4.00	31	0.73	3.88	86	0.82	0.416
19.7	…Address violent/antisocial behavior in schools	4.00	53	0.78	3.94	31	0.89	3.95	86	0.83	0.730

**Attitudes toward teaching martial arts**											
**Question**	**Field**	**Female**			**Male**			**Total**			
		**Mean**	***N***	**SD**	**Mean**	***N***	**SD**	**Mean**	***N***	**SD**	Significance
Q20.1	I believe children and/or adolescents in the class(es) I teach would enjoy participating in martial arts training	3.96	53	0.83	4.19	31	0.70	4.02	86	0.80	0.197
Q20.2	I believe children and adolescents in general would enjoy participating in martial arts training	4.02	53	0.82	4.35	31	0.61	4.13	86	0.76	0.051
Q20.3	I would enjoy teaching martial arts to students	3.66	53	1.04	3.94	31	1.15	3.76	86	1.07	0.263

**Future plans**											
**Question**	**Field**	**Female**			**Male**			**Total**			
		**Mean**	***N***	**SD**	**Mean**	***N***	**SD**	**Mean**	***N***	**SD**	Significance
Q22.1	In the future, I would be interested in learning how to incorporate martial arts in a classroom setting	4.08	52	0.86	4.03	31	0.95	4.04	85	0.91	0.826
Q22.2	I would be interested in attending professional development/accreditation to learn how to teach basic martial arts techniques to my students	3.92	52	1.03	3.97	31	0.95	3.92	85	1.00	0.844
Q22.3	In future, I would be interested in a martial arts‐based program that also improved NSW KLA syllabus outcomes	3.87	52	0.86	4.06	31	0.85	3.93	85	0.86	0.311
Q22.4	If martial arts were introduced into the NSW curriculum, I would outsource the instruction, rather than teach it myself	3.65	52	0.97	3.65	31	0.92	3.64	85	0.95	0.968
Q22.5	In the future I would be comfortable incorporating martial arts in my own classroom	3.52	52	0.98	3.87	31	0.89	3.64	85	0.95	0.105
Q22.6	In the future I plan on participating/training (or continuing) in some form of martial art (e.g., Boxing, Kickboxing, Wrestling, Jujitsu, Karate, Taekwon‐Do etc)	3.21	52	1.16	3.48	31	1.31	3.31	85	1.24	0.328

**Most beneficial martial arts topics for children and adolescents**											
**Question**	**Field**	**Female**			**Male**			**Total**			
		**Mean**	***N***	**SD**	**Mean**	***N***	**SD**	**Mean**	***N***	**SD**	Significance
Q23.1	Movement/parkour type activities (e.g., rolling, jumping, vaulting, falling safely)	4.22	51	0.70	4.26	31	0.63	4.21	84	0.68	0.784
Q23.2	Learning how to dodge and block attacks	4.22	51	0.67	4.39	31	0.62	4.27	84	0.65	0.252
Q23.3	Learning how to punch	3.73	51	1.08	3.84	31	1.00	3.73	84	1.08	0.637
Q23.4	Learning how to kick	3.73	51	1.04	3.87	31	0.99	3.74	84	1.05	0.534
Q23.5	Grappling/wrestling (e.g., how to safely escape if tackled or grabbed)	3.92	51	0.94	4.26	31	0.86	4.05	84	0.90	0.107
Q23.6	Teamwork/team building activities among classmates	4.53	51	0.61	4.55	31	0.72	4.52	84	0.65	0.899
Q23.7	Wellbeing/ethical discussions at the end of each session, focussing on topics such as respect, integrity, self‐control and so forth	4.49	51	0.61	4.55	31	0.68	4.50	84	0.63	0.689
Q23.8	Olympic martial arts: Judo, Taekwon‐Do and Karate	3.75	51	1.07	3.97	31	0.88	3.80	84	1.04	0.333
Q23.9	Learning katas/forms/poomsae (set movement routines)	3.73	51	1.00	3.77	31	1.09	3.70	84	1.06	0.837
Q23.10	Meditation/mindfulness	4.41	51	0.78	4.29	31	1.07	4.37	84	0.89	0.555
Q23.11	Learning how to use various traditional weapons (e.g., swords, Bo staff, Kali sticks)	3.08	51	1.32	3.03	31	1.33	3.04	84	1.32	0.879

*Note*: Two participants who selected “Other” for sex so were not included in the subgroup analysis due to small numbers.

Abbreviations: *N*, number of responses; SD, standard deviation.

Two statistically significant differences were found in his section. The first can be seen in Q18.3: Martial arts training can improve muscular fitness in children and adolescents (*p* = 0.039). While this was the highest mean score for both groups in this section, (score≥4.2 out of 5), practicing teachers rated this higher (score = 4.58) than preservice teachers (score = 4.23).

The second significant difference was found in question 19.1: *Martial arts training can improve mental health in children and adolescents* (*p* = 0.027). Again, both rated this section highly (score ≥ 4 out of 5), and following this trend, teachers rated this question higher (score = 4.46) than preservice teachers (score = 4.08). Although not statistically significant in most areas, the mean score for the practising teacher group was higher in all 10 questions in this section, indicating that they may place more value on MA and the benefits they believe it can provide children and adolescents than preservice teachers do. The attribute with the lowest perceived benefit was not the same for both groups. The lowest mean score for teachers was found in Q19.7: *Martial arts training can…address violent/antisocial behavior in schools* (mean = 4.04). The lowest mean score in this section for preservice teacher was found in Q19.6: *Martial arts training can…help children and adolescents get along better with other people in their life (e.g., family, classmates, friends)* (mean = 3.78).

##### Attitudes toward teaching martial arts

There were no significant differences between groups in this section. The scores were quite similar in all questions. All participants agreed that: the students they teach would enjoy participating in MA (scores≥ 4 out of 5); that children and adolescents in general would enjoy participating in MA (scores≥ 4 out of 5). Q20.3 was the question with the lowest mean in this section for both groups, however, both groups still indicated they would enjoy teaching MA to students (scores≥ 3.4 out of 5).

##### Future plans

A statistically significant difference was seen in Q22.3: *In the future, I would be interested in a martial arts‐based program that also improved NSW KLA syllabus outcomes* (*p* = 0.014). In the top three mean scores given by both groups, two intergroup commonalities were identified. Both groups had Q22.2 and Q22.3 in their top three highest mean scores (scores≥ 3.77 out of 5), indicating a general interest in attending professional learning for MA teaching among both groups.

##### Most beneficial martial arts topics

A statistically significant difference between both preservice and practicing teacher participants was found in “movement/parkour type activities (e.g., rolling, jumping, vaulting, falling safely)” (*p* = 0.000). Both groups' five highest rated topics were the same. *Teamwork/team building activities among classmates* and *wellbeing/ethical discussions at the end of each session, focussing on topics such as respect, integrity, self‐control etc*. being the top two subjects (respectively) for both groups. *Meditation/mindfulness*, *movement/parkour type activities (e.g., rolling, jumping, vaulting, falling safely)*, and *learning how to dodge and block attacks* were also found in the top five in both groups, but varied slightly in rankings. Both groups ranked *learning how to use various traditional weapons (e.g., swords, bo staff, kali sticks)* the lowest (Teachers = 2.88; Preservice teachers = 3.10).

#### 3.4.2 Comparison of means—Sex

##### Beliefs

Table 3 shows a statistically significant difference between male and female participants' beliefs relating to inclusion of MA training in primary school settings. While both males and females had a positive skew toward martial arts (scores≥3.58 out of 5), males rated this question higher (*F* = 3.59, *M* = 4.00; *p* = 0.017). There was a generally high mean score in favor of providing students in both primary (female = 4.04; male = 4.17) and high‐school (female = 4.16; male = 4.33) settings with the opportunity to try MA training. The lowest total mean was found in Q17.6: *I believe the NSW Department of Education promotes martial arts as an acceptable in‐school sport*, (female = 3.07; male = 2.77). Although both groups scored above 2.5, skewing in the direction of positive belief towards this question, there was a level of uncertainty around whether the NSW Department of Education promotes MA as an acceptable in‐school sport in both groups. Both participant groups indicated that they would strongly to very strongly (scores ≥4.13 out of 5) support the introduction of self‐defense or MA (of any kind) into the curriculum, however, participants were less confident in their ability to teach it themselves (female = 3.05; male = 3.32).

##### Perceived benefits of martial arts training

A statistically significant difference (*p* = 0.001) was seen in question 18.3: *Martial arts training can improve muscular fitness in children and adolescents*. Male participants responded more strongly to this question, but both groups responded strongly to this question (scores ≥4.14 out of 5). Although generally statistically nonsignificant, the “male” means for 9 out of 10 of these questions were higher than “female.” All means were skewed in a way that indicates participants believe MA training can provide children and adolescents with physiological and psychological, and social benefits.

##### Attitudes toward teaching martial arts

No statistically significant differences were seen in this section, however, intergroup difference in Q20.2: *I believe children and adolescents in general would enjoy participating in martial arts training* approached statistical significance (*p* = 0.051), with the “male” group again scoring slightly more positively toward this belief. Although still in favor (scores≥3.65 out of 5), all respondents were less positive toward teaching MA to students themselves.

##### Future plans

There were no statistically significant differences between groups. A trend was found, with Q22.1, Q22.2, and Q22.3 all rating the highest among “female” and “male” groups. All six questions returned means skewed in favor of MA (mean≥3.21).

##### Most beneficial martial arts topics for children and adolescents

No statistically significant differences were found in this section. Each gender shared a number of commonalities among the five highest mean scored topics. Q23.6: *Teamwork/team building activities among classmates*; Q23.7: *Wellbeing/ethical discussions at the end of each session, focussing on topics such as respect, integrity, self‐control and so forth*; Q23.2: *Learning how to dodge and block attacks*; Q23.10: *Meditation/mindfulness*; Q23.1: Movement/Parkour type activities (e.g., rolling, jumping, vaulting, falling safely); and Q23.5: *Grappling/wrestling (e.g., how to safely escape if tackled or grabbed)* were all shown to be included in the topics with the highest mean scores among each group. Q23.6: *Teamwork/team building activities among classmates*; and Q23.7: *Wellbeing/ethical discussions at the end of each session, focussing on topics such as respect, integrity, self‐control and so forth* both scored as the highest, or second‐highest mean score given by each group.

#### 3.4.3 Qualitative feedback

##### Concerns for the inclusion of martial arts in (my) school

There were two trends in the areas of concern given by respondents. The most common concern related to a perceived risk of injury. Several respondents voiced their concerns of *Legal issues i.e., kids getting hurt*, and *how [they] would control it to ensure no one gets hurt*. The other common concerns were related to the *promot*[ion] *of violence in the school setting*, and the *incorrect use* in the playground, practicing unsupervised.

##### Suggestions for increasing the implementation of martial arts in schools

Again, two distinct trends emerged in these suggestions. The first being professional learning and resources. Respondents suggested to *give teachers and preservice teachers training* and to provide *Easy to use resources including a website with videos to follow step by step*. The second trend was a suggestion for MA to have a *focus… on self‐defense rather than fighting*, as well as a promotion of a *… teamwork component &… mental wellbeing aspect & how it can positively impact* participants.

## 4 DISCUSSION

This questionnaire provided new insight into a selection of Australian teachers' (practicing and undergraduate) views and beliefs relating to MA and the implementation of such training in school settings. While the data indicates high levels of support for MA in school settings, some respondents showed their concerns for the *safety* of students, and the potential of students *getting hurt*. Interestingly, the Australian Institute of Health and Welfare Australian sports injury data (collected) 2012–2013 ranked MA (labeled combative sports and included Aikido, Chi Kung, Judo, Jujitsu, Karate, Kendo, Kick‐boxing, Ninjitsu, Taekwon‐Do, Tai Chi, and Martial arts [other]) as the 10th most commonly associated with hospitalized sport‐related facial fractures, shoulder dislocations, and injury to the cervical spine.^21^ Sports such as Rugby union, Australian Rules football, Rugby league, Soccer, and Cycling respectively were associated with greater injury rates on these areas than MA.^21^ Of note, in other injury categories, such as sport‐related cruciate ligament injury or head injuries combative sports did not rank in the top 10.^21^ Additionally, in a study based on major trauma cases and deaths in the state of Victoria, Australia over a 10‐year period, the highest three frequencies were in cycling, motor sports and equestrian activities,^22^ with neither Combative Sports nor MA of any kind ranking at all. It is also important to note that these results are from competitive and/or leisure and community based sport. School‐based activities are usually at a foundational and noncompetitive development levels, and thus present a lower risk of injury.

As stated by a multitude of researchers,^23^, ^24^, ^25^, ^26^, ^27^ one of the main barriers preventing people from taking up MA as a hobby, or welcoming MA into the school environment, is the misconception of traditional MA. Traditional MA training often becomes misinterpreted and associated with the fighting skills and violence that are glorified and propagated in movies, television shows and other popular culture.^10^ The “martial artist” stereotype depicted in these films finds themselves in several situations, in which he or she has to defeat an opponent (or a whole room full of opponents) using acrobatics, choreographed kick combinations, and weapons. The kicking and screaming of Bruce Lee is often mentioned as an example of the MA stereotype. This seemingly works in two ways. The first being that his superior fighting skills highlight the violence typically associated with MA, which leads to the misrepresentation that is being discussed. The second stereotype being that his signature screams and sounds, along with his small stature and regular attack of the larger opponent's groin seem to undercut his legitimacy as a tough, heterosexual warrior,^24^ and project the martial artist as a dirty fighter (someone that fights using unfair or frowned upon techniques) with a high pitched scream. Both stereotypes are counterproductive in attaining MA acceptance in school settings. The first highlighting a largely misrepresented emphasis on violence, and the second displaying a lack of normative hetero‐masculinity,^24^ by the male practitioner, although the actuality being that these activities are open to participants of all skill and fitness levels, sex and age. This may influence those who plan school PDHPE programs, and in turn, deter the incorporation of MA training.

In addition to concerns of the perceived danger of MA training, many respondents also showed a lack of knowledge relating to the current NSW Department of Education School Sport Unit rules and regulations for the safe delivery of MA training in schools. One independent schools‐based respondent noted that *it is [my] understanding that these types of activities are banned*. Additionally, the majority of teacher respondents indicated that they did not believe the NSW Government views MA training as an acceptable school sport. An investigation into the NSW Department of Education School Sport Unit website proves this belief to be misinformed, and provides a S*port safety guidelines* document for MA. With the majority of respondents having less than 4 years teaching experience, this gap in knowledge may be attributed to a lack of exposure throughout their preservice teacher training, or professional development for qualified teachers. A recommendation for this may be to include a brief introduction to MA and various simple MA activities at university for preservice primary school and PDHPE specialist teachers.

### 4.1 Strengths, limitations, and suggestions for future studies

It should be noted that none of the school principals agreed to invite their teachers to participate, with all responses coming through social media or undergraduate university courses. To the authors knowledge this is the first survey study to investigate preservice and in‐service teachers' perceptions of MA in Australia. None of the 22 school Principals invited to complete the survey accepted the invitation. As a consequence, this sample size is too small to make conclusive generalizations about the views and beliefs of the target population. However, it provides an insight into the views of a diverse group of teachers and preservice teachers. This study provides a strong case for further investigation into this topic across all states of Australia. A larger sample size is required to form conclusive generalizations on this topic. One solution to the sampling issue would be to request that the same questionnaire could be sent out by the NSW Department of Education, to all teachers. Furthermore, this survey could be sent out to other states and countries, with comparisons made between each.

## 5 PERSPECTIVE

This study indicates a high level of support for MA in school settings in a sample of current teachers and preservice teachers from Australia. Many participants did not realize that “NSW Government School Sport” had a protocol for teaching MA. The results indicate that teachers and preservice teachers view MA as a worthwhile activity, with a range of benefits including physiological and psychological, and support the inclusion of MA in school settings. These findings may provide support to advocate for more teacher training and professional development to enable the safe delivery of MA, and/or high quality PDHPE (and school sport) programs that draw from MA to deliver key outcomes in PDHPE. Furthermore, respondents indicated their support for MA content in school programs that incorporate teamwork, wellbeing/ethical discussions, learning how to dodge and block attacks, meditation/mindfulness, movement/parkour activates, as well as grappling/wrestling. This allows for the creators of future intervention, PDHPE, or sport programs to provide programs that incorporate the input provided by current and preservice teachers. This may in turn assist with school engagement with sports medicine‐based interventions, as such interventions are now able to reflect content that the teachers have shown to value and view as beneficial to students. Although a larger study is recommended to strengthen these conclusions, this study provides Australian school authorities with a baseline to further support teachers in their efforts to incorporate MA in school settings, through the provision of preservice teacher training, professional development for current teachers, or resources and program development.

## AUTHOR CONTRIBUTIONS

**Louis Burt**: Conceptualization; data curation; formal analysis; investigation; methodology; project administration; resources; writing—original draft; writing—review & editing. **Nicholas Riley**: Conceptualization; formal analysis; investigation; methodology; project administration; supervision; writing—review & editing. **Narelle Eather**: Conceptualization; formal analysis; investigation; methodology; project administration; supervision; writing—review & editing.

## ETHICS STATEMENT

This study was approved by the Human Research Ethics Committee of [The University of Newcastle]. Approval number: H‐2019‐0057.

## TRANSPARENCY STATEMENT

The lead author Louis Burt affirms that this manuscript is an honest, accurate, and transparent account of the study being reported; that no important aspects of the study have been omitted; and that any discrepancies from the study as planned (and, if relevant, registered) have been explained.

## Data Availability

Data available on request from the authors.
